# Calcium Polyphosphate Nanoparticles Act as an Effective Inorganic Phosphate Source during Osteogenic Differentiation of Human Mesenchymal Stem Cells

**DOI:** 10.3390/ijms20225801

**Published:** 2019-11-18

**Authors:** Luan Phelipe Hatt, Keith Thompson, Werner E. G. Müller, Martin James Stoddart, Angela Rita Armiento

**Affiliations:** 1 AO Research Institute Davos, 7270 Davos Platz, Switzerland; phelipe.hatt@aofoundation.org (L.P.H.); keith.thompson@aofoundation.org (K.T.); martin.stoddart@aofoundation.org (M.J.S.); 2 Institute for Physiological Chemistry, University Medical Center of the Johannes Gutenberg University, 55131 Mainz, Germany; wmueller@uni-mainz.de

**Keywords:** osteogenic differentiation, mesenchymal stem cells, β-glycerolphosphate, inorganic polyphosphate, Ca-polyphosphate nanoparticles

## Abstract

The ability of bone-marrow-derived mesenchymal stem/stromal cells (BM-MSCs) to differentiate into osteoblasts makes them the ideal candidate for cell-based therapies targeting bone-diseases. Polyphosphate (polyP) is increasingly being studied as a potential inorganic source of phosphate for extracellular matrix mineralisation. The aim of this study is to investigate whether polyP can effectively be used as a phosphate source during the in vitro osteogenic differentiation of human BM-MSCs. Human BM-MSCs are cultivated under osteogenic conditions for 28 days with phosphate provided in the form of organic β-glycerolphosphate (BGP) or calcium-polyP nanoparticles (polyP-NP). Mineralisation is demonstrated using Alizarin red staining, cellular ATP content, and free phosphate levels are measured in both the cells and the medium. The effects of BGP or polyP-NP on alkaline phosphatase (ALP) activity and gene expression of a range of osteogenic-related markers are also assessed. PolyP-NP supplementation displays comparable effects to the classical BGP-containing osteogenic media in terms of mineralisation, ALP activity and expression of osteogenesis-associated genes. This study shows that polyP-NP act as an effective source of phosphate during mineralisation of BM-MSC. These results open new possibilities with BM-MSC-based approaches for bone repair to be achieved through doping of conventional biomaterials with polyP-NP.

## 1. Introduction

Bone-marrow-derived mesenchymal stem/stromal cells (BM-MSCs) are multipotent cells able to differentiate into bone-lineage-committed osteoblasts and eventually, into terminally differentiated osteocytes. This feature has fueled intense research on the use of BM-MSCs as a source of bone-forming osteoblasts with which to treat bone-related conditions.

A culture system enabling the in vitro osteogenic differentiation of adult human BM-MSCs has been reported as early as 1997 [1]. The described osteogenic cocktail contains 100 nM dexamethasone (Dexa), 10 mM β-glycerolphosphate (BGP) and 50 µM l-ascorbic acid-2-phosphate (AA2P) and it is typically supplemented to Dulbecco’s Modified Eagle’s Medium containing 1 g/L glucose (LG-DMEM) medium containing 10% fetal bovine serum (FBS). The use of this differentiation cocktail induces cell morphological changes, increases the activity of alkaline phosphatase (ALP) and promotes the formation of mineralised extracellular matrix.

Within the osteogenic cocktail, BGP provides an organic source of phosphate. On the cell membrane, ALP converts BGP to free phosphate (P_i_) which is then used by the cells to sustain the collagen deposition during the early stage of differentiation and to form hydroxyapatite during mineralisation. Schäck and colleagues have demonstrated that the phosphate source influences the quality of mineralised matrix produced by hBM-MSCs [2]. In particular, a high concentration of BGP (10 mM) causes non-physiological fluctuations of free P_i_ levels in media, while administration of inorganic phosphate in the form of Na_2_HPO_4_ maintains the P_i_ levels in the medium at a stable level. Furthermore, using an inorganic phosphate source results in a calcium to phosphate ratio in the mineralised matrix that is closer to that of carbonated hydroxyapatite present in human bone (1.63) [3]. In rat calvaria cells, BGP is rapidly converted to P_i_ during the early stages of mineralisation and can be replaced by P_i_ with formation of a mineralised tissue indistinguishable from the one obtained when using BGP [4]. In an organ culture of parietal bones from rat calvaria, BGP and high doses of P_i_ (6 mM) caused ectopic and dystrophic calcification in the periosteum [5].

Inorganic P_i_ has been proposed to work not only as a building block for hydroxyapatite formation but most importantly, as a signaling molecule regulating cell function and differentiation [6]. In murine alveolar bone cells, P_i_ increases the gene expression of type I collagen, ALP, bone sialoprotein (BSP) [7] and osteopontin [8]. In human dental pulp cells, P_i_ regulates the expression of bone morphogenetic protein-2 at both the gene and protein level [9].

Inorganic polyphosphates (polyP) are linear polymers of orthophosphate that can reach hundreds of phosphates in length. The role of polyP in cellular physiology has mostly been studied in prokaryotic organisms. The presence of polyP in eukaryotic cells is established but little is known about its synthesis and metabolism and, therefore, its biological function in mammalian cells remains to be elucidated. PolyP are present at higher levels in osteoblast-like cells than in cells derived from soft tissue, suggesting a role for polyP in osteoblast function [10]. Exogenous polyP can act as a source of inorganic P_i_ for mineralisation in murine MC3T3-E1 osteoblast-like cells and can support bone formation in both a canine [11] and a rat alveolar bone defect [12]. The addition of polyP to cultures of human dental pulp-derived cells or MSCs results in cell calcification and increased type I collagen gene expression and molecule maturation [13]. In BM-MSCs derived from patients affected by osteoarthritis or rheumatoid arthritis, polyP increase gene expression of early markers of osteoblast formation such as ALP and type I collagen, and a late marker of mineralisation, BSP [14].

Recently, Müller et al. have described the fabrication of a polyP-based material in the form of calcium polyP nanoparticles (polyP-NP) that can be hydrolysed in cultures of SaOS-2 osteoblast-like cells [15]. In SaOS-2 cells cultured in the presence of an osteogenic cocktail including BGP, the supplementation of polyP-NP causes translocation of ALP on the cell membrane and increases adenosine triphosphate (ATP) production and release, suggesting a role of polyP as a cellular metabolic fuel [16]. Although SaOS-2 cells display increased hydroxyapatite deposition when cultured in classical osteogenic media containing BGP-supplemented with additional polyP-NP [17], the effect of polyP-NP on the osteogenic differentiation in the absence of other phosphate sources awaits clarification. Additionally, polyP-NP treatment has been demonstrated to induce short-term proliferative effects on hBM-MSCs (≤3 days) [18,19] and to increase expression of chondrogenic and osteogenic transcription factors (*Sox9* and *Runx2*, respectively), in murine BM isolated from femoral explants [18]. However, the effects of polyP-NP-supplementation during the long-term in vitro osteogenic differentiation of human BM-MSCs remain unknown.

The aim of this study is to investigate if polyP-NP can effectively be used as an alternative phosphate source to BGP during in vitro osteogenic differentiation of human BM-MSCs. Should polyP-NP be capable of supporting osteogenic differentiation of BM-MSCs, this would permit the potential doping of biomaterials for tissue purposes aimed at bone restorative approaches.

## 2. Results

### 2.1. Inorganic PolyP-NP can Function As an Effective phosphate Source for Matrix Mineralisation during Osteogenic Differentiation of Human BM-MSCs

The capacity of inorganic polyP-NP to serve as an effective phosphate source for matrix mineralisation during the osteogenic differentiation of human BM-MSCs is assessed using Alizarin red staining after 14 and 28 days of culture. To demonstrate this, a classical differentiation media containing BGP (Osteogenic (BGP)) is compared to an identical media composition but where 10 mM BGP is replaced with 30 μg/mL polyP-NP (Osteogenic (polyP-NP)). As an additional control, basal medium is also used to assess spontaneous osteogenic differentiation of hBM-MSCs in the absence (Osteocontrol) or presence of 30 µg/mL polyP-NP (Osteocontrol + polyP-NP).

**Figure 1:**
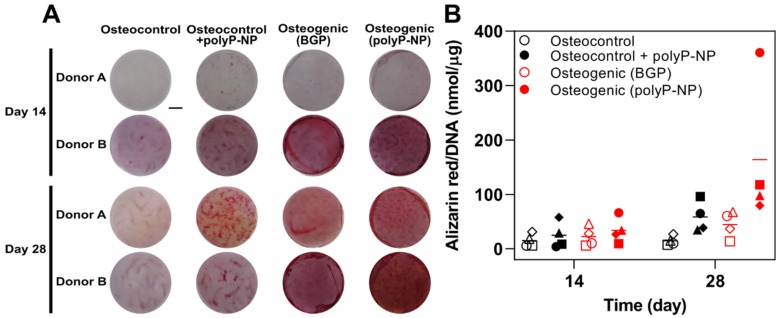
Alizarin red staining and quantification. (**A**) Representative images of Alizarin red staining of human bone-marrow-derived mesenchymal stem/stromal cells (BM-MSCs) at day 14 (upper panel) and day 28 (lower panel) from two independent donors are indicated for the four different treatments. Scale bar: 3 mm; (**B**) Quantification of Alizarin red staining normalised to DNA content for all donors. Individual data points shown are the mean of two technical replicates for each individual donor: donor A (□), donor B (Δ), donor C (◊) and donor D (o). The mean of the four independent donors within each group is indicated by the horizontal bar.

The classical BGP-containing osteogenic differentiation medium induces robust mineral deposition in monolayer cultures, particularly at day 28, although this is highly variable between the four independent donors under investigation (Figure 1). Substitution of BGP with polyP-NP results in an overall trend for increased levels of matrix mineralisation compared to the classical osteogenic differentiation cocktail. However, the mere addition of polyP-NP to osteocontrol medium significantly increases Alizarin red staining in these non-osteogenic cultures by day 28, but not on day 14. This indicates that the capacity of polyP-NP to enhance matrix mineralisation is increased only during the later stages of osteogenic differentiation of BM-MSCs and is not merely an artifact of the Alizarin red staining protocol. However, the approximate fold increase in Alizarin red staining between the classical BGP-containing osteogenic media and osteocontrol medium in comparison to the polyP-NP-containing osteogenic medium and the polyP-NP-containing osteocontrol medium is 2.3- and 2.8-fold, respectively. This indicates that polyP-NP can effectively act as a phosphate source, and thus can function as a substitute for BGP in osteogenic differentiation media, but relatively insensitive assays such as Alizarin red may suffer from increases in background staining, thus requiring appropriate negative controls.

### 2.2. PolyP-NP Treatment Results in Comparable Effects on ALP Activity to BGP during Osteogenic Differentiation of hBM-MSCs

**Figure 2:**
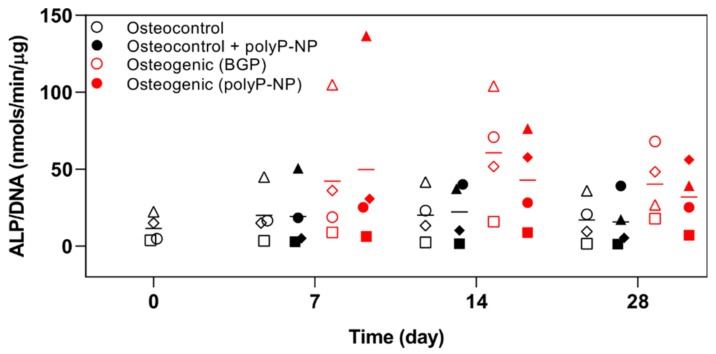
Alkaline Phosphatase (ALP) activity between treatment groups over time. Human BM-MSCs are cultured in osteocontrol media ± polyP-NP, or osteogenic medium containing either BGP or polyP-NP, for 7, 14 or 28 days. ALP activity in cell lysates is then determined and normalised to DNA content. Data are shown as the mean of two technical replicates for each individual donor: donor A (□), donor B (Δ), donor C (◊) and donor D (o). The mean of the four independent donors within each group is indicated by the horizontal bar.

The classical BGP-containing osteogenic media consistently increased ALP activity on day 7 and 14, although marked heterogeneity in individual donors is observed (Figure 2). Interestingly, the donor with the highest degree of BGP-induced mineralization (Donor B), as assessed by Alizarin red staining (Figure 1), also has the highest basal level of ALP activity in osteocontrol medium, together with the most rapid BGP-induced increase (peak at day 7 and 14) in ALP activity. Comparable responses with polyP-NP-containing osteogenic media for inducing increases in ALP activity are also observed throughout the 28-day period. Conversely to the observed effects of polyP-NP alone for inducing increased background staining in the Alizarin red assay, there is no effect on ALP activity when polyP-NP alone are added to osteocontrol medium. Therefore, polyP-NP supplementation induces a similar ALP activity to that induced by classical BGP-containing osteogenic media in human BM-MSCs.

### 2.3. Osteogenic Differentiation of BM-MSCs, Supported by Either BGPor polyP-NP, Is Associated with Increased Intracellular Levels of ATP at Early TIME Points

**Figure 3:**
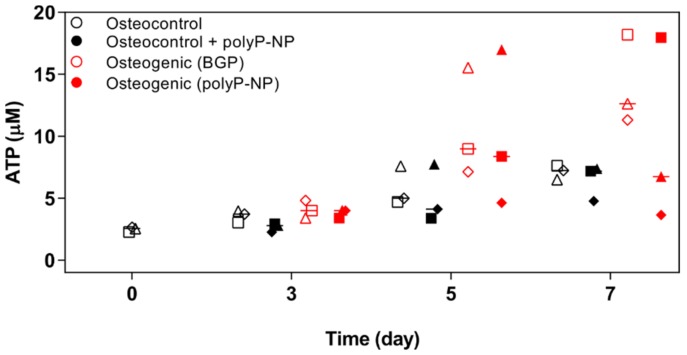
Intracellular ATP content between treatment groups over time. Human BM-MSCs are cultured in osteocontrol media ± polyP-NP, or osteogenic medium containing either BGP or polyP-NP, for 0, 3, 5 or 7 days. ATP content in cells is then determined. Individual data points indicated are the mean of two technical replicates for each independent donor: donor A (□), donor B (Δ) and donor C (◊). The mean of the three independent donors within each group is indicated by the horizontal bar.

PolyP-NP have previously been shown to enhance mineralisation of osteoblast-like SaOS-2 cells when added to a classical osteogenic differentiation medium including BGP [17]. Short-term treatment with polyP-NP has also demonstrated to induce ATP production within 3 h in human umbilical vein endothelial cells (HUVECs). We, therefore, want to determine if a similar enhanced metabolic response is evident when polyP-NP are cultured with BM-MSCs under either normal or osteogenic conditions. Under the same experimental conditions as reported for Alizarin red staining and ALP activity, we have measured ATP levels in BM-MSCs at days 0, 3, 5 and 7. BGP-supplemented osteogenic media increase ATP content at days 5–7 (Figure 3). PolyP-NP-containing osteogenic medium induces a comparable response to BGP-containing osteogenic media with increased ATP content at day 5, but this difference is not observed at day 7. No additional benefit of polyP-NP supplementation for increasing ATP production is evident when cells are cultured in osteocontrol medium alone. Taken together, this suggests that the enhanced mineralisation evident with polyP-NP-supplemented osteogenic medium compared to BGP-supplemented osteogenic medium (Figure 1) is not due to the provision of metabolic fuel and thereby, increasing ATP levels in BM-MSCs are observed.

### 2.4. BGP Results in High Free Phosphate Fluctuations in the Medium

**Figure 4:**
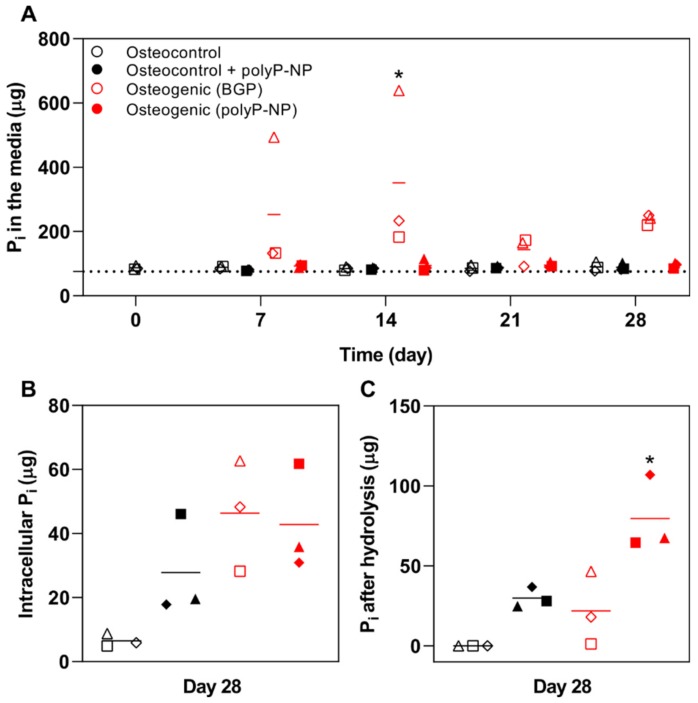
Quantification of free phosphate (P_i_) levels. Human BM-MSCs are cultured in osteocontrol media ± polyP-NP, or osteogenic medium containing either BGP or polyP-NP, for 28 days. (**A**) Conditioned media samples are collected at days 7, 14, 21 and 28 and P_i_ levels in the media samples are determined. The dashed line at 86 µg/mL represents the detected P_i_ concentration in LG-DMEM without additional supplementation. Two-way ANOVA is performed; *(*p* < 0.001 vs. osteogenic; *p* < 0.001 vs. osteocontrol + polyP-NP; *p* < 0.05 vs. osteocontrol (polyP-NP)); (**B**) Intracellular P_i_ concentrations in cell lysates of the different treatment groups at day 28; (**C**) Total potential P_i_ in the cell lysates is assessed following acid hydrolysis. One-way ANOVA is performed; *(*p* < 0.001 vs. osteocontrol; *p* < 0.05 vs. osteocontrol + polyP-NP; *p* < 0.05 vs. osteogenic (BGP)). Data are shown as the mean of two technical replicates for each individual donor: donor A (□), donor B (Δ) and donor C (◊). The mean of the three independent donors within each group is indicated by the horizontal bar.

We then attempt to determine if the observed differences in mineralisation due to polyP-NP supplementation of osteogenic medium is due to the enhanced provision of free P_i_, thereby driving the mineralisation process. Interestingly, only BGP-supplemented osteogenic medium is found to have enhanced levels of P_i_ in conditioned medium (day 14: *p* < 0.001 vs. osteocontrol; *p* < 0.001 vs. osteocontrol + polyP-NP; *p* < 0.05 vs osteogenic (polyP-NP)) taken weekly during the differentiation process (Figure 4A). PolyP-NP supplementation to either osteocontrol or osteogenic medium does not result in P_i_ levels above those already detected in osteocontrol medium alone. When cell and extracellular matrix lysates from day 28 cultures are also analysed, P_i_ levels are increased in osteogenic cultures, with no differences observed between BGP- and polyP-NP-supplemented osteogenic medium (Figure 4B). Of further note, polyP-NP-supplemented osteocontrol medium also demonstrated increased levels of P_i_ compared to levels in osteocontrol medium-treated cells. When the total potential P_i_ pool is determined, by acid-hydrolysing the cell lysate to liberate P_i_ from bound polyP in the cells and extracellular matrix, polyP-supplementation of either osteocontrol or osteogenic medium results in increased levels of potential P_i_, which is significant for polyP-NP-supplemented osteogenic media (Figure 4C). This demonstrates that polyP-NP supplementation provides an abundance of potential phosphate for mineralisation to occur.

### 2.5. PolyP-NP Induce Similar Changes to BGP-Containing Osteogenic Media in the Expression of BM-MSC-Relevant Transcription Factors

**Figure 5:**
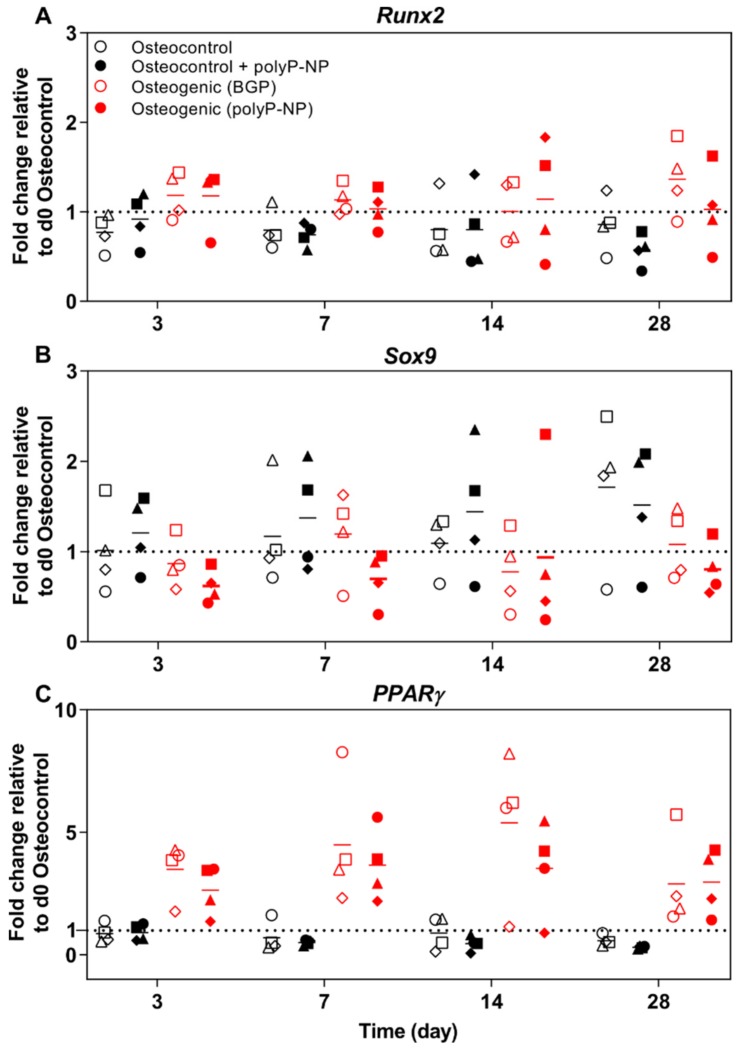
Gene expression of transcription factors involved in the differentiation of BM-MSCs. Human BM-MSCs are cultured in osteocontrol media ± polyP-NP, or osteogenic medium containing either BGP or polyP-NP, for 28 days. Fold changes in *Runx2* (**A**), *Sox9* (**B**) and *PPARγ* (**C**) expression at day 3, 7, 14, 21 and 28 are calculated according to the ΔΔ*C*t method using *RPLP0* as the endogenous calibrator and the osteocontrol at day 0 as the normaliser. Individual data points shown are the mean of two technical replicates for each individual donor: donor A (□), donor B (Δ), donor C (◊), and donor D (o). The mean of the four independent donors within each group is indicated by the horizontal bar.

We then determine the expression of a range of differentiation-relevant transcription factors for BM-MSCs related to osteogenesis (*Runx2*), chondrogenesis (*Sox9*) and adipogenesis (*PPARγ*). Changes in gene expression are presented as the fold-change relative to the housekeeping gene (*RPLP0*) and the osteocontrol group at day 0. Treatment of hBM-MSCs with BGP-containing osteogenic media results in an overall trend for increased *Runx2* expression compared to osteocontrol media at days 3, 7 and 28 (Figure 5A). Similar responses are also obtained with polyP-NP-containing osteogenic media. Regarding the chondrogenic differentiation marker *Sox9*, there is a trend for decreased expression at day 3 for both BGP- and polyP-NP-supplemented osteogenic media, suggesting that the ratio of *Runx2:Sox9* is more informative for determining the osteogenic differentiation potential (Figure 5B). Suppression of *Sox9* expression by polyP-NP-supplemented osteogenic media compared to poly-NP-supplemented osteocontrol media is also evident at day 7. At day 28, both BGP- and polyP-NP-containing osteogenic media induce marked reductions in *Sox9* expression compared to the respective osteocontrol media. Interestingly, there is a pronounced expression of the adipocyte differentiation marker *PPARγ* in BM-MSCs treated with either the BGP- or polyP-NP-containing osteogenic media (Figure 5C) from days 3–28, which again supports the capacity of polyP-NP to induce comparable responses to BGP-containing osteogenic media in BM-MSC differentiation protocols.

### 2.6. PolyP-NP-Supplementation of Ostegenic Medium Supports Expression of Early, Intermediate and Late Osteogenic Markers Comparable to BGP-Supplemented Osteogenic Media

In addition to transcription factor expression, we additionally investigate the expression of other early-(collagen 1A1; *Col1A1*), mid-(ALP; *ALPL*) and late-(BSP; *IBSP*) stage osteogenic marker genes. BSP is a component of mineralised tissue and is encoded by the *IBSP* gene. 

**Figure 6:**
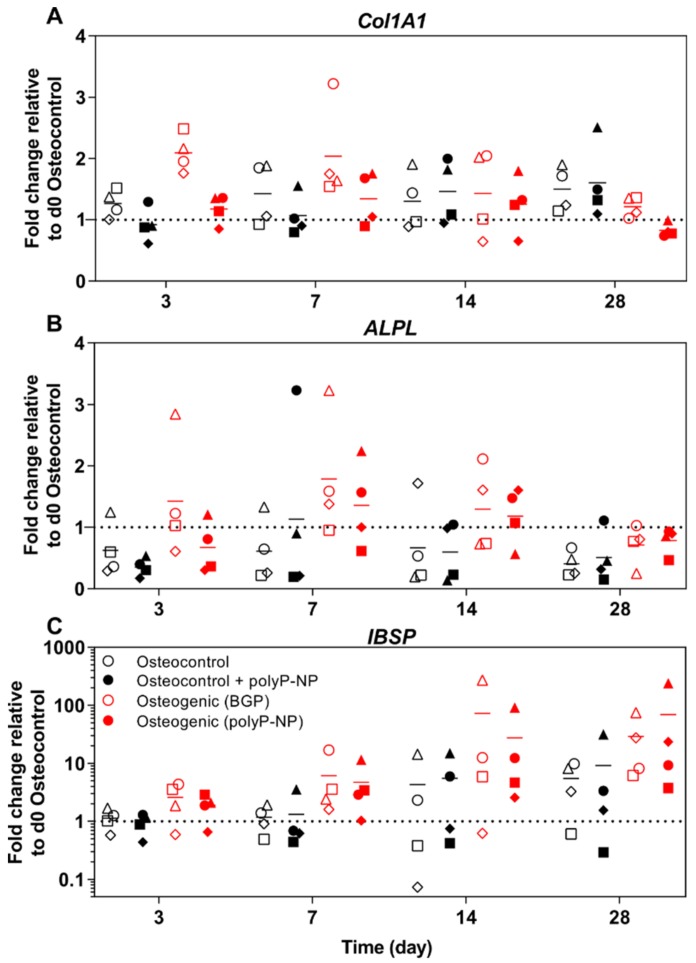
Gene expression of osteogenic markers during differentiation of BM-MSCs. Human BM-MSCs are cultured in osteocontrol media ± polyP-NP, or osteogenic medium containing either BGP or polyP-NP, for 28 days. Fold changes in *Col1A1* (**A**), *ALPL* (**B**) and *IBSP* (**C**) expression at days 3, 7, 14 and 28 are calculated according to the ΔΔ*C*t method using *RPLP0* as the endogenous calibrator and the osteocontrol at day 0 as the normaliser. Individual data points shown are the mean of two technical replicates for each individual donor: donor A (□), donor B (Δ), donor C (◊) and donor D (o). The mean of the four independent donors within each group is indicated by the horizontal bar.

Regarding *Col1A1* expression, BGP-supplemented osteogenic media increases *Col1A1* expression at days 3 and 7, relative to osteocontrol media (Figure 6A). Although there is a trend for PolyP-NP-supplemented osteogenic media to increase *Col1A1* expression at day 3, no differences are evident at day 7, relative to the polyP-NP-supplemented osteocontrol media. Additionally, there is no effect of polyP-NP supplementation on *Col1A1* expression in osteocontrol media. BGP-containing osteogenic media increases *ALPL* expression rapidly by day 3, when compared to osteocontrol media, which was maintained at days 7 and 14 (Figure 6B). Similarly, polyP-NP-supplemented osteogenic media also increases *ALPL* expression compared to polyP-NP-supplemented osteocontrol media, although this is only observed at days 7 and 14. Expression of the late-stage osteogenic marker BSP is also increased in response to BGP- and polyP-NP-supplemented osteogenic media, when compared to the respective osteocontrol media, but this is only evident at days 14 and 28 (Figure 6C). 

Taking the findings of gene expression data together, polyP-NP appears to be as effective as BGP for inducing representative changes in BM-MSCs indicative of osteoblastic differentiation.

## 3. Discussion

The much-heralded promise of MSC-based approaches for the treatment of musculoskeletal disorders continues to remain tantalizingly out of reach in terms of its clinical translation. Therefore, strategies aimed at more faithfully recapitulating the process of MSC differentiation in vivo may provide a means for enhancing MSC-based therapies, particularly in the context of tissue-engineering approaches aimed at restoring lost bone tissue. With this in mind, we have investigated whether polyP-NP could substitute for the classical phosphate source, BGP, during the in vitro osteogenic differentiation of BM-MSCs. We have determined that polyP-NP can effectively replace BGP in osteogenic differentiation medium based on their similar efficacy for stimulating mineralised matrix deposition, ALP activity and osteogenic gene expression in human BM-MSC cultures. Due to their stable nature, the addition of polyP-NP may, therefore, provide a novel means for inducing osteogenic effects in biomaterials utilised in musculoskeletal disorders.

Previous studies have demonstrated that polyP-NP can induce proliferative effects on human BM-MSCs at 3 days [18,19] and can increase both mineralisation as well as *Sox9* and *Runx2* expression in murine bone marrow isolated from femoral explants after 7 days [18]. Our study demonstrates that polyP-NP-supplementation effectively supports the osteogenic differentiation of human BM-MSCs over a longer 28-day period, typically used to assess human BM-MSC osteogenic differentiation, and provides a detailed temporal characterisation of mineralisation, ALP production and alterations in osteogenic gene expression in response to polyP-NP supplementation throughout the culture period.

Regarding osteogenic differentiation, mineralised matrix production is a classical marker for osteogenic potential of MSCs, which assesses the extent of mineral deposition within the extracellular matrix, of which type I collagen is a principle component. Therefore, classical osteogenic differentiation media for MSCs typically includes ascorbic acid (or a more stable derivative such as ascorbic acid-2-phosphate), to promote type I collagen fibril assembly, a source of phosphate, to promote mineral deposition, and Dexa to activate Wnt/β-catenin signaling [20]. Expression of the important osteogenic differentiation factor *Runx2*/*Cbfa1* is another classical marker for MSC differentiation, although we do not observe any marked differences in *Runx2* expression induced by either osteogenic media containing BGP or polyP-NP. This reflects a known difference in the osteogenic responses between rodent and human BM-MSCs, whereby *Runx2* appears to be constitutively expressed in human MSCs and is not altered during osteogenic differentiation, with its increased activity due to enhanced *Runx2* phosphorylation [21]. Consistent with our study, a further study detailing no increase in *Runx2* expression during osteogenic differentiation of human BM-MSCs has also demonstrated a concomitant decrease in *Sox9* expression, thus suggesting that the ratio of *Runx2:Sox9* expression appears to be a more reliable indicator of osteogenic differentiation in human BM-MSCs [22].

Although polyP-NPs perform, in most respects, equivalent to BGP in terms of supporting osteogenic differentiation of human BM-MSCs, some important differences are evident. A striking difference can be observed in the extent of free P_i_ present in the osteogenic media when supplemented with BGP compared to polyP-NP. BGP-containing osteogenic media typically contains markedly higher levels of free P_i_ (Figure 4) throughout the differentiation period, with levels apparently dictated by the extent of ALP activity present in the cells, consistent with a previous study [2]. Despite a previous study reporting that ALP activity is not proportional to resulting mineralisation [23], at least under our experimental protocol, increased ALP activity appears to correlate with ultimate mineralisation at day 28 (as assessed by Alizarin red staining) in the case of BGP-supplemented osteogenic media, but not with polyP-NP-supplemented osteogenic media. Interestingly, free P_i_ levels are not increased following polyP-NP supplementation to either osteocontrol or osteogenic media, indicating that ALP activity does not liberate free P_i_. However, following acid hydrolysis of the cell monolayer, there are higher levels of free P_i_ in cells treated with either the polyP-NP-supplemented osteocontrol or osteogenic media, indicating that a large potential pool of free P_i_ exists in the form of polyP, either stored intracellularly or sequestered in the extracellular matrix. Therefore, the supportive effects of polyP-NP on the osteogenic differentiation process of BM-MSCs does not appear to be mediated via the provision of extracellular free P_i_, as is evidently the case with BGP supplementation.

One mechanism previously proposed for the supportive effects of polyP-NP supplementation on osteogenic gene expression (*Col1* and *ALPL*) in SaOS-2 osteosarcoma cells is via the provision of metabolic energy following clathrin-mediated endocytosis of the polyP-NP [17]. Uptake of polyP-NPs by SaOS-2 cells has been confirmed using transmission electron microscopy. The authors have additionally demonstrated that polyP-NP-treated HUVECs rapidly increase intracellular ATP levels within 3 h. After assessing ATP levels during our osteogenic differentiation protocol at 3, 5 and 7 days, we do not observe any increase in ATP levels following polyP-NP treatment of BM-MSCs under either osteocontrol or osteogenic conditions. Although we cannot presently exclude the possibility that rapid increases in cellular ATP levels occur in the early hours following polyP-NP treatment, this does not appear to be the mechanism by which polyP-NP support osteogenesis during long-term osteogenesis differentiation protocols necessary for human BM-MSCs. Further studies are required.

MSC biology is plagued by issues of high inter-donor variability, particularly in the case of long-term osteogenic and chondrogenic differentiation assays. To cite one example regarding osteogenic differentiation, in a screening of 19 individual MSC donors, up to 53-fold differences in ALP gene expression have been observed [2]. Given our reported findings with variabilities in Pi levels following BGP-supplementation and the impact of different ALP activities from individual donors, this suggests that inorganic phosphate sources such as polyP-NP or Na_x_H_3-x_PO_4_ [2] may provide a means for reducing such variability in free Pi levels, thereby resulting in less inter-donor variability in osteogenesis assays of human BM-MSCs.

PolyP has been shown to improve osteogenic differentiation of pre-osteoblastic MC3T3 cells when doped onto hydroxyapatite-coated surfaces [24] and when incorporated into hyaluronic acid hydrogels [25]. Additionally, polyP-NPs have also been shown recently to be capable of acting as a biomimetic periosteal coating of allogeneic bone samples, facilitating SaOS-2 cell adhesion and mineral deposition [26]. Thus, polyphosphate supplementation of biomaterials appears to be an attractive approach to provide osteoconductive cues in a variety of bone-focused tissue engineering methodologies.

Currently, it is unknown whether polyP-NPs can effectively substitute for BGP in supporting the osteogenesis of MSCs isolated from different sources such as adipose tissue [27] or perinatal tissue such as cord blood [28]. However, given the ease with which to obtain such tissue, in contrast to BM-MSCs, the use of such MSCs may provide a rich cell source for future tissue engineering approaches. Therefore, should polyP-NPs be capable of supporting osteogenesis in a range of MSCs isolated from different tissues, polyP-NP may provide a stable means for inducing osteogenic responses in tissue engineering strategies aimed at restoring lost bone.

## 4. Materials and Methods

Human bone marrow aspirates are obtained with informed consent of all donors and with full approval from the Ethics Committee of the University of Freiburg Medical Centre (EK-Freiburg: 135/14, 25 March 2014) and the ethical commission of Graubünden (KEK-ZH-NR: 2016-00141). All reagents are purchased from Sigma-Aldrich (St. Louis, MO, USA) unless otherwise stated.

### 4.1. Preparation of Calcium-PolyP-NP

PolyP-NP are prepared as described previously [15]. Briefly, 10 g of Na-polyphosphate (Na-polyP of average chain of 40 phosphate units) are dissolved in 500 mL distilled water and the pH is adjusted to 10 with 1 M NaOH solution. A solution of 20 g/L CaCl_2_ dihydrate in distilled water is then added dropwise to the Na-polyP solution in a CaCl_2_ to polyP weight ratio of 2:1. After 4 h of stirring, the precipitated nanoparticles are collected and washed twice with ethanol. The particles are then dried at 60 °C, sterilised by ethylene oxide and stored at room temperature.

### 4.2. Cell Isolation and Culture

Human BM-MSCs are isolated from bone marrow aspirates and cryopreserved as previously described [29]. Upon thawing, BM-MSCs are seeded at a density of 3 × 10^3^ cells/cm^2^ in T300 flasks for culture expansion and cells from all donors are used up to passage 5. The expansion medium consists of α-MEM (Gibco, Carlsbad, CA, USA) supplemented with 10% (*v*/*v*) Sera Plus bovine serum (PAN-Biotech, Aidenbach, Germany), 100 U/mL Penicillin, 100 µg/mL Streptomycin (PEN/STREP) (Gibco) and 5 ng/mL basic fibroblast growth factor (Fitzgerald Industries, North Acton, MA, USA). Cells are cultured under standard conditions of 37 °C with 5% CO_2_ and 90% humidity. Donor details of the BM-MSCs used in this study are as follows: Donor A—48-year old male, spine vertebral body aspirate; Donor B—57-year old female, spine vertebral body aspirate; Donor C—73-year old female, spine vertebral body aspirate; and Donor D—53-year old male, femoral cancellous bone.

### 4.3. Osteogenic Differentiation

At passage 3 to 4, cells are harvested using 0.05% Trypsin-EDTA (Gibco) and seeded on Thermanox™ coverslips (Nunc, Rochester, NY, USA) placed within 24-well plates at a density of 1.5 × 10^4^ cells/cm^2^. For the first 24 h, cells are cultured in osteocontrol medium consisting of LG-DMEM (Gibco) supplemented with 10% (*v*/*v*) fetal bovine serum (FBS) (Gibco) and 1% (*v*/*v*) PEN/STREP. At this point (day 0), cells are switched into four different culture media compositions, as detailed in Table 1. Briefly, two groups are cultured with osteocontrol medium in the absence or presence of 30 µg/mL polyP-NP, respectively. The third group consists of osteocontrol medium additionally supplemented with the classic osteogenic cocktail including 10 nM Dexa, 50 µl/mL AA2P and 10 mM BGP. The fourth group is the classic osteogenic cocktail but with the BGP replaced by 30 µg/mL polyP-NP. Cells are kept under standard culture conditions for 28 days with media changed three times per week.

### 4.4. RNA Isolation and qRT-PCR

Cells are harvested for gene expression analysis at day 0, 3, 7, 14 and 28 by incubation with Tri Reagent supplemented with 0.5% (*v*/*v*) Polyacryl Carrier (Molecular Research Center, Inc., Cincinnati, OH, USA) at room temperature, then stored at −80 °C for at least 24 h. For total RNA isolation, cell lysates are thawed at room temperature, then 0.1 mL of 1-Bromo-3-chloropropane (BCP) per 1 mL Tri Reagent is added to each sample. After incubation at room temperature for 15 min and centrifugation (12,000× *g* at 4 °C) for 15 min, the aqueous phase is transferred into fresh tubes. RNA is precipitated using 0.5 mL isopropanol at room temperature with gentle agitation for 10 min. Samples are then centrifuged (12,000× *g* at 4 °C) for 8 min, the supernatant removed, and the RNA pellet is washed and centrifuged three further times with 1 mL of ice-cold 75% ethanol. The ethanol is then removed, the pellet left to air-dry and is then resuspended in 20 µL of DEPC-treated water and incubated at 65 °C for 15 min. RNA concentrations and purity are measured using the NanoDrop™ 1000 spectrophotometer (Thermo Scientific, Waltham, MA, USA). Two-step quantitative reverse transcriptase PCR (qRT-PCR) is carried out using 500 ng of RNA in a 40 µL reaction. cDNA is diluted 1 to 5 using 1X Tris-EDTA and stored at −20 °C until further use.

Real-time PCR is performed using a QuantStudio™ Flex Real-Time PCR System (Applied Biosystems, Waltham, MA, USA). Primer sequences used are listed in Table 2. Data analysis is performed using the ΔΔ*C*t method using *RPLP0* as an endogenous normalizer and day 0 samples as a calibrator.

### 4.5. Alizarin Red Staining and Quantification

Cells are cultured as previously described in Section 4.3 and then assessed for Alizarin red staining at days 14 and 28. Cells are washed three times with phosphate-buffered saline (PBS), fixed with buffered 4% formalin solution for 20 min at room temperature and subsequently washed three times with distilled water. The fixed cells are then incubated with 0.2 mL of 40 mM Alizarin red solution (pH 4.1–4.3) at room temperature for 1 h with gentle agitation. Subsequently, cells are repeatedly washed with distilled water until no further discoloration of the water is evident.

After imaging, the amount of bound Alizarin red in the cell monolayer is quantified. A quantity of 0.4 mL of 10% (*v*/*v*) acetic acid is added to each well and the plates are incubated for 30 min at room temperature on an orbital shaker. The cell monolayers are then transferred into Eppendorf tubes (Eppendorf, Hamburg, Germany), vortexed and incubated at 85 °C for 10 min. Samples are then cooled on ice for 5 min and centrifuged at 20,000× *g* for 15 min at room temperature. The supernatant is collected, then the pH adjusted to 4.1–4.5 with 10% (*v*/*v*) ammonium hydroxide. The absorbance is measured at 405 nm using the Victor3™ plate reader (PerkinElmer, Waltham, MA, USA) and the concentration of Alizarin red calculated by comparison with a standard curve prepared from the stock Alizarin red solution.

### 4.6. Quantification of ALP Activity and DNA Content

Cells are cultured in the respective osteocontrol or osteogenic media and then harvested for assessment of alkaline phosphatase (ALP) activity at day 0, 3, 7, 14 and 28. Cell monolayers are washed with PBS and then lysed in 0.1% Triton X-100 for 2 h at 4 °C. Alkaline buffer solution, substrate solution (25 mg/mL phosphate substrate in 1 mM diethanolamine buffer containing 0.5 mM MgCl_2_, pH 9.8) and deionised water are added to the cell lysate, and the enzymatic reaction is carried out at 37 °C for 15 min. The reaction is stopped by addition of 0.1 M NaOH solution. The absorbance is read at 405 nm using the Victor3™ plate reader. ALP activity is normalised to DNA content, following determination using the CyQuant Assay (Thermo Fisher, Waltham, MA, USA), according to the manufacturer’s instructions. Briefly, the cell lysate is incubated with CyQuant Gr dye for 5 min, protected from the light. The emitted fluorescence is measured at 530 nm and DNA content was calculated using a standard curve generated using calf thymus DNA.

### 4.7. Quantification of ATP Content

Cells are cultured in either osteocontrol media (in the presence or absence of polyP-NP) or the respective osteogenic media for 0–7 days before assessment of ATP content using the Luminescent ATP Detection Assay Kit (Abcam Ab113849, Cambridge, MA, USA). The assay was performed according to the manufacturer’s instructions Luminescence was measured using a Victor3^™^ plate reader.

### 4.8. Phosphate Quantification

Levels of free P_i_ are assessed in conditioned media samples were harvested once per week. Following collection, media is stored at −20 °C prior to analysis. Additionally, free P_i_ content is also assessed in cultures at day 28. Cells are washed with Tris buffered saline (TBS) solution, incubated at 4 °C for 10 min, then harvested using a cell scraper and stored at −20 °C. Upon thawing, the cells are lysed with three cycles of sonication as follows: 30 s sonication, 10 s pause and 10 s sonication. The cell lysate is then centrifuged at 13,000× *g* for 10 min at 4 °C and the supernatant is collected. To assess total potential phosphate content, the remaining cell pellet is subsequently hydrolysed using 1 M HCl for 24 h at 37 °C. The acidified cell lysate is then centrifuged at 13,000× *g* for 10 min and the supernatant is collected. P_i_ concentrations in the samples are measured using the QuantiChrom Phosphate Assay Kit (DPI-500, BioAssay Systems, Hayward, CA, USA) according to the manufacturer’s instructions.

### 4.9. Statistics

All experiments are performed on three or four independent donors, with measurements being conducted in duplicates in order to reduce methodological variability. A two-way analysis of variance (ANOVA) is performed for experiments across multiple time points. A one-way ANOVA is performed for experiments with one time point. Only significant differences between the two osteogenic treatments are marked with an asterisk (* *p* < 0.05). Analyses are carried out using the software package GraphPad Prism 8 (GraphPad Software Inc., San Diego, CA, USA).

## Figures and Tables

**Table 1:** Media composition of the four experimental groups.

	Osteocontrol	Osteocontrol + polyP-NP	Osteogenic (BGP)	Osteogenic (polyP-NP)
LG-DMEM ^1^	+	+	+	+
PEN/STREP	+	+	+	+
FBS	+	+	+	+
Dexa	-	-	+	+
AA2P	-	-	+	+
BGP	-	-	+	-
polyP-NP	-	+	-	+

^1^ LG-DMEM: low glucose (1 g/L) DMEM, PEN/STREP: Penicillin/Streptomycin, FBS: fetal bovine serum, Dexa: dexamethasone, AA2P: L-ascorbic acid 2-phosphate sesquimagnesium salt hydrate, BGP: β-Glycerolphosphate disodium salt hydrate, polyP-NP: polyphosphate nanoparticles. The “+” indicates the presence and the “-“ indicates the absence of the supplement in the medium.

**Table 2:** Primers/probes used for qRT-PCR.

**Gene**	**Forward**	**Reverse**	**Probe**
*COL1A1*	5′-CCC TGG AAA GAA TGG AGA TGA T-3′	5′-ACT GAA ACC TCT GTG TCC CTT CA-3′	5′-CGG GCA ATC CTC GAG CAC CCT-3′
*RPLP0*	5′-TGG GCA AGA ACA CCA TGA TG-3′	5′-CGG ATA TGA GGC AGC AGT TTC-3′	5′-AGG GCA CCT GGA AAA CAA CCC AGC-3′
*RUNX2*	5′-AGC AAG GTT CAA CGA TCT GAG AT-3′	5′-TTT GTG AAG ACG GTT ATG GTC AA-3′	5′-TGA AAC TCT TGC CTC GTC CAC TCC G-3′
**Gene**	**Assay on Demand Ref No. (Applied Biosystems)**		
*ALPL*	Hs00758162_m1		
*IBSP*	Hs0017320_m1		
*PPAR-γ*	Hs00234592_m1		
*SOX9*	Hs00165814_m1		

## Abbreviations

AA2P	L-ascorbic acid 2-phosphate sesquimagnesium salt hydrate
ALP	Alkaline phosphatase
ANOVA	Analysis of variance
ATP	Adenosine triphosphate
BCP	1-Bromo-3-chloropropane
BGP	Β-Glycerolphosphate disodium salt hydrate
BSP	Bone sialoprotein
Dexa	Dexamethasone
FBS	Fetal bovine serum
hBM-MSCs	Human bone marrow-derived mesenchymal stem cells
HUVEC	Human umbilical vein endothelial cells
LG-DMEM	Low glucose (1 g/L) Dulbecco’s Modified Eagle’s Medium
PBS	Phosphate-buffered saline
PEN/STREP	Penicillin/Streptomycin
Pi	Free phosphate
polyP-NP	Polyphosphate nanoparticles
*PPARγ*	Peroxisome proliferator-activated receptor gamma
qRT-PCR	Quantitative reverse transcription PCR
*Runx2*	Runt-related transcription factor 2
*Sox9*	Sex determining region Y box 9
TBS	Tris buffered saline
